# Pharmacovigilance teaching and learning: a mixed cross-sectional analysis of the Portuguese public higher education system

**DOI:** 10.1186/s12909-023-04963-1

**Published:** 2024-01-03

**Authors:** Margarida Perdigão, Anabela Afonso, Sofia de Oliveira-Martins, Manuel José Lopes, Ana Margarida Advinha

**Affiliations:** 1 Pharmacovigilance Regional Unit of the Central and North Alentejo, University of Évora, Evora, Portugal; 2 School of Nursing S. João de Deus, Department of Nursing, University of Évora, Evora, Portugal; 3 School of Sciences and Technology, Department of Mathematics, University of Évora, Evora, Portugal; 4 Research Center for Mathematics and Applications – CIMA, Evora, Portugal; 5 Faculty of Pharmacy of the University of Lisbon, Lisbon, Portugal; 6 CHRC – Comprehensive Health Research Centre, Evora, Portugal; 7 School of Health and Human Development, Department of Health and Medical Sciences, University of Évora, Evora, Portugal

**Keywords:** Adverse drug reactions, Healthcare courses, Healthcare professionals, Higher education system, Pharmacovigilance

## Abstract

Pharmacovigilance stands out for its importance in obtaining existing knowledge about medicine and patient safety and should be recognized as a continuous line of study. It constitutes a highly relevant component in the activities of health professionals, with spontaneous notification of suspected adverse drug reactions being its main emphasis. The underreporting that persists can be overcome through continuous professional development programs, reinforcing theoretical and practical knowledge in the curricular plans of health courses. As a result, more educated professionals will also allow citizens to recognize the importance of pharmacovigilance. The main objective of this study was to describe and characterize the teaching-learning process of pharmacovigilance in Portugal, analyzing the knowledge, perceptions and attitudes of students and health professionals. In total, ninety-three curricular unit forms of the seventeen healthcare courses included were analyzed, among which only three referred to pharmacovigilance as mandatory and thirty-nine did not address any keywords. The questionnaire applied was answered by 650 participants, both students (62%) and professionals (38%). Approximately 84.4% of the students and 54.7% of the professionals affirmed that they had never spontaneously reported an adverse drug reaction. Only 24.6% of the students and 17.8% of professionals referred to the existence of specific course content dedicated to pharmacovigilance in their coursework. In view of these results, it is evident that there is a need for a wider reflection regarding the further training and constant update of practicing professionals as well as in diverse health institutions, investing in the creation of an academic curriculum that integrates pharmacovigilance in healthcare courses.

## Key findings

• Teaching pharmacovigilance in healthcare courses is needed.

• Greater reflection on professional training is needed.

• Community-based programs on pharmacovigilance are recommended.

## Introduction

The development of new medicines involves clinical studies that aim to assess not only efficacy but also safety and quality. However, when marketing authorization (MA) is approved, the information available is insufficient [1] due to the numerous limitations of clinical trials [2]. Therefore, it is necessary to have postmarketing monitoring of the drug and a continuous assessment of its risks and benefits, given that although they are safe, they are not risk-free. This is where pharmacovigilance programs shine, promoting patient safety, preventing adverse drug reactions (ADRs) and thus improving clinical practice [3].

In recent years, the evolution of pharmacovigilance and its growing importance as a fundamental element of individual and public health has been widely described to demonstrate its relevant activity and outcomes [3]. Society in general and patients have become more aware and involved, and the concept of pharmacovigilance has also evolved and expanded [4]. Currently, pharmacovigilance is firmly based on strong scientific principles and is considered a clinical discipline, as it contributes to an ethic of safety and serves as an indicator of the standards of healthcare practice in a country. However, as a curricular unit, it needs to be further developed to significantly contribute to individual and public health and to clinical practice in this area, considering the expectations of students in higher education, trained professionals, and the population in general [3].

Notably, the pharmacovigilance system only works if health professionals, marketing authorization holders and citizens report ADRs, enriching it with a real, innovative, and pragmatic perspective [5]. Spontaneous reporting of suspected ADRs is one of the main sources of information in pharmacovigilance, as well as a right and a duty, so it should progressively become a habit of professionals and citizens, thus integrating joint work [4]. The underreporting that persists may be related to the lack of knowledge about the process of reporting suspected ADRs [3, 6], and Portugal is not an exception [4, 7–9]. For this reason, an aspect to be highlighted is the strengthening of the participation of health professionals and patients, as well as the general population in pharmacovigilance systems worldwide. In view of this, it is crucial to promote and disseminate pharmacovigilance activities among students and health professionals [4].

The main objective of this study was to describe and characterize the teaching-learning process of pharmacovigilance in Portugal. To do so, the knowledge, perceptions and attitudes of students and health professionals were analyzed, as well as the main difficulties that were identified by the professionals regarding spontaneous notification.

## Methods

**Fig. 1:**
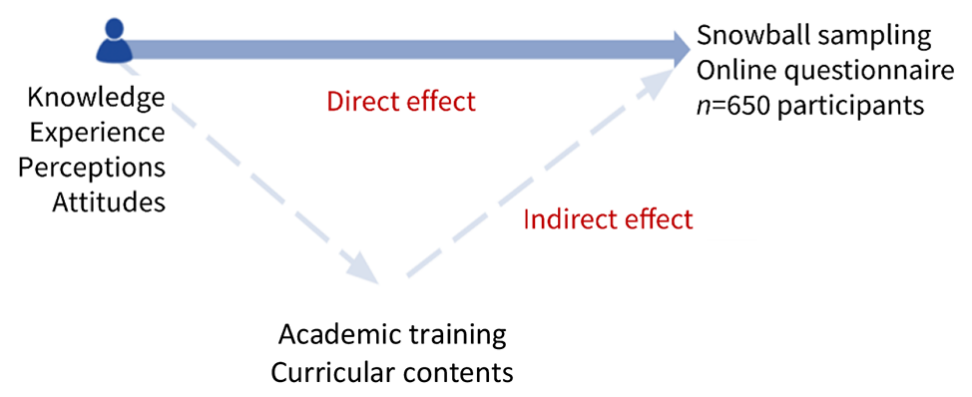
Direct and indirect analyses

A mixed cross-sectional [10, 11] design was used. In this sense, a mixed curriculum analysis method was adopted, consisting of a direct analysis and an indirect analysis, as shown in Fig. 1.

The direct analysis corresponded to a study on the results obtained from the response to a questionnaire by the respondents (whether they were students or health professionals) as potential holders of knowledge, experiences, perceptions, and attitudes in ​​pharmacovigilance. The questionnaire was built based on a bibliographic revision on this topic, aiming to explore issues identified in other studies [9, 12–19]. In the indirect analysis, a systematic, explicit, and clear review was carried out, with the search for keywords in the curricular unit forms of the curricular plan of courses in the health area from ​​public institutions of the Portuguese higher education system approved by the Directorate General for Higher Education (*DGES*) (Table 1), with the aim of understanding the syllabus, that is, the academic training on pharmacovigilance effectively given to higher education students. The criteria for this preselection included the approach to health courses from which future professionals should have, during their professional practice, a closer relationship and contact directly with patients and, with the management of their drug regimen, have a privileged position to detect, act on and report any suspected ADRs.

**Table 1:** Health courses analyzed

Health courses analyzed according to the DGES website
Audiology
Biomedical Pharmacy
Dental Medicine
Dietetics and Nutrition
Medical Imaging and Radiotherapy
Medicine
Nursing
Nutrition Sciences
Occupational Therapy
Oral Hygiene
Orthoptics
Pharmacy
Physiology Clinic
Physiotherapy
Psychology
Speech Therapy

Concerning the indirect analysis of the curricular unit forms/descriptions, a systematic search using 44 terms/keywords was performed (Table 2).

**Table 2:** Terms/keywords searched in the indirect analysis

Terms and keywords identified in the syllabus of the analyzed health courses	
Specific	General
Active and passive	(Good) habits
Administration	(Information) sources
Adverse drug reaction	Adherence
Adverse effects	Assessment
Benefit-risk	Classification
Causality	Detection
Clinical trials	Marketing authorization/MA
Contraindications	Mistakes
European Medicines Agency/EMA	Portugal
INFARMED, I.P.	Precautions
Interactions	Prevention/preventive
Measures (minimization)	Problems
*MedDRA* dictionary	Patient/user(s)
Medication	Public health
Medicines	Rational
Monitoring	Regulatory/regulation
National Pharmacovigilance System	Secondary
Pharmacoepidemiology	Therapeutic
Pharmacovigilance	Use
Risk	Vigilance
Safety/safe	World Health Organization/WHO
Sign (safety)	
Spontaneous notification	

Obtention of the approved and recently dated curricular unit forms (the school years 2019–2020 and 2020–2021) was carried out through the official websites of higher education institutions.

To blind this analysis, a codification system was carried out through the attribution of a code to the course, public higher education institution, curricular unit, year, and semester corresponding to each form.

The direct analysis consisted of the description of the answers obtained through the application of a questionnaire, intending to characterize the following:

- Sociodemographic data.
- Individual experiences regarding ADRs and pharmacovigilance.
- Students’ basic knowledge about pharmacovigilance.
- Perceptions of teaching and learning on the part of students about the subject area of pharmacovigilance.
- Perceptions of all respondents about the importance of pharmacovigilance and overall training in this area.
- Potential determinants (barriers and facilitators) to spontaneous reporting of suspected ADRs by health professionals.

The questionnaire had specific questions for students and healthcare professionals. The sections relating to sociodemographics, individual experiences regarding ADRs and pharmacovigilance, perceptions about the importance of pharmacovigilance and perceptions regarding overall training in pharmacovigilance were common to both groups. The questions regarding knowledge about pharmacovigilance and perceptions of teaching-learning were included only in the questionnaire for students, and questions referring to the main determinants (barriers and facilitators) of spontaneous notification of suspected ADRs were only for health professionals. A pretest was carried out among ten students and ten health professionals with the same conditions as those applied for the final questionnaire.

A snowball sampling methodology was used, and the dissemination of the questionnaire to students and health professionals took place on social networks and via electronic mail sent to institutional contacts, namely, pharmacies, health centers, hospitals, and universities, among others. Data collection was carried out by self-completion of the electronic questionnaire and took place for one month between the 6th of May and the 7th of June 2021. A total sample of 650 participants was obtained, among the 30,477 students [20] who were enrolled in the courses covered by this study and the 84,568 health professionals, which is the national total number of health professionals present in the 2018 Ministry of Health Report [21].

There is no reliable information on the size of the total number of the population that was involved in this study. The student population was the students who were enrolled in the courses covered by this study, that is, higher education students present on the DGES statistics page and the total national number of professionals in the Ministry of Health reports.

A Microsoft Excel® database was created to analyze the courses in the field of health, respective institutions, and descriptive data from each course. The database also contained the curriculum plan (school years 2019–2020 and 2020–2021) about the programmatic contents in pharmacovigilance: whether there was a specific pharmacovigilance curricular unit or whether it was addressed in another related unit. This database was built using information available on the *DGES* website, more precisely in the health section. The systematization and descriptive analysis of the keywords collected in the curricular unit forms was also developed using this database matrix. Univariate and bivariate descriptive analyses of the data obtained were carried out using graphical and tabular representations and summary measures. Absolute frequencies and summary statistical measures were calculated for numerical variables, and categorical variable analysis of frequencies (counts and percentages) was used. The data obtained were processed and analyzed using the Statistical Package for the Social Sciences (SPSS) v.27 and Microsoft Office Excel®. For ethical reasons, to guarantee the confidentiality of the data and the anonymization of the institutions, considering the general objective of this study—to analyze the national pharmacovigilance teaching framework (not individualizing the institutions that teach the different courses) - the higher education institutions and respective curricular units were coded.

Regarding data collected via the online questionnaire, participants provided informed consent using an initial question, in which the respondents confirmed that they understood the purpose of this study and agreed to participate. The participation of all respondents was voluntary. For the present study, only grouped data were of interest, so the responses to the questionnaire were anonymous. Subsequently, all data were collected and processed by computer, treated, and stored in a safe place, following data protection legislation, with access only by the research team, thus guaranteeing their anonymity and confidentiality; the data could not reveal the identity of the participant, nor was any data collected disclosed to third parties. The present study was approved by the Ethics Committee of the University of Évora (ID: 21,028).

## Results

Indirect analysis was used to conduct a systematic search of keywords that was based on the bibliographic research on pharmacovigilance in the international literature and on the frequency of certain types of words that appear in the plan of all the curricular unit forms obtainable from the seventeen courses that were selected for this study, resulting in a total of forty-three identified keywords.

This collection consisted of identifying curricular units dedicated to pharmacovigilance or its integration into other units of a more general spectrum.

In total, ninety-three (*n =* 93) curricular unit forms that addressed content related to pharmacovigilance, either in specific curricular units dedicated to this topic or through other curricular units that addressed the topic of the seventeen healthcare courses included were analyzed (Audiology; Nutrition Sciences; Pharmaceutical Sciences; Nutrition and Dietetics; Nursing; Pharmacy; Biomedical Pharmacy; Clinical Physiology; Physiotherapy; Oral Hygiene; Medical Image and Radiotherapy; Medicine; Dental Medicine; Orthoptics and Vision Sciences; Psychology; Speech Therapy and Occupational Therapy), in which only three referred to a mandatory pharmacovigilance curricular unit (Pharmacy; Pharmaceutical Sciences; Biomedical Pharmacy) and thirty-nine (42%) did not address any keywords, belonging to four courses (Orthoptics and Vision Sciences; Speech Therapy; Occupational Therapy and Audiology).

The top five keywords identified were *medicines*, *pharmacovigilance*, *adverse drug reactions*, *safety*, *adverse effects*, and *spontaneous notification* belonging to five courses (Pharmacy; Nursing; Pharmaceutical Sciences; Medicine and Biomedical Pharmacy).

Briefly, in Portugal, the lack of early and consistent exposure to pharmacovigilance in the studied health courses was highlighted, with only 3 mandatory and 1 optional curricular unit dedicated exclusively to this area belonging to courses in Pharmaceutical Sciences, Pharmacy, and Biomedical Pharmacy. Thus, in the other courses, pharmacovigilance is not part of the curriculum, not even as an option, with the teaching of this topic being visible in other units. However, in some higher education courses in ​​health, there is not even an approach to syllabi related to the theme. Pharmacy had the highest number of related terms present in its curricular unit forms, 129 in total, followed by the Nursing course (*n =* 126), Pharmaceutical Sciences (*n =* 83) and Medicine (*n =* 21).

The indirect analysis was carried out systematically to investigate the current skills of pharmacovigilance in students from various areas of health, namely Medicine, Nursing, Pharmacy/Pharmaceutical Sciences and Dentistry. The courses on Biomedical Pharmacy (*n =* 19), Oral Hygiene, and Medical Imaging and Radiotherapy follow, with 18 keywords identified for each, Dentistry (*n =* 14), Dietetics and Nutrition (*n =* 12), Nutrition Sciences (*n =* 7), and Clinical Physiology and Psychology, with 5 referenced keywords. Finally, Physiotherapy had only 2 keywords, and Audiology, Orthoptics and Vision Sciences, Speech Therapy, and Occupational Therapy had no related terms mentioned in the curricular unit forms.

**Fig. 2:**
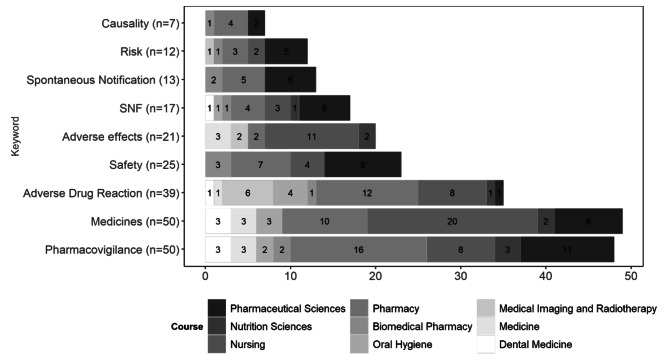
Absolute frequency of keywords in the courses that presented these keywords most frequently

Summarizing the results obtained through the indirect analysis, a total of 129, 126, 83 and 21 keywords were verified in the Pharmacy, Nursing, Pharmaceutical Sciences and Medicine courses, respectively. Figure 2 presents the frequency of the keywords by health course.

Direct analysis - The questionnaire applied was answered by 650 participants, 403 students (62%) and 247 professionals (38%) (Table 3). All participants who accessed the questionnaire responded.

**Table 3:** Sociodemographic data

	Students	Healthcare Professionals
Respondents	83.4%	80.2%
Age (years)	21.2 (± 3.2)	36.7 (± 11.1)
Never reported an ADR (%)	84.4	54.7
Reported having addressed pharmacovigilance in their degree (%)	24.6	17.8

### Individual experiences relative to adverse drug reactions and pharmacovigilance

Both students and health professionals reported that they more often perceived a suspected ADR in closer individuals, more precisely, in family, friends and/or users/patients than in themselves.

Approximately 84.4% of the students and 54.7% of the professionals affirmed that they had never made a spontaneous ADR notification, and a minority of students (9.7%) and 41.7% of health professionals had already reported a suspected ADR. Only 24.6% of the students and 17.8% of professionals reported specific course content dedicated to pharmacovigilance in their degrees; however, the same proportion (65%) in both groups of respondents affirmed that the topic was addressed in other curricular units of the course.

When asked about the presence of a specific curricular unit dedicated to pharmacovigilance in the course, 24.6% of students and 17.8% of professionals attended a specific curricular unit dedicated to teaching it, and 4.7% of students and 5.3% of professionals revealed that the curricular unit was optional. However, the largest group, corresponding to 54.8% of students and 68% of practicing professionals, reported that the curricular unit in question did not exist in their course, but the majority (64.7% and 63.7%) responded that the theme was addressed in other curricular units.

### Basic knowledge of pharmacovigilance

Approximately 81.9% of the students reported having heard of pharmacovigilance and were knowledgeable of the importance of spontaneous reporting of suspected ADRs. The majority (66.7%) of the students were familiar with its relevance, and 67.2% were aware of its concept and purpose. Likewise, 77.4% reported that there was content related to pharmacovigilance in their course, with respondents from the Pharmacy, Oral Hygiene, Pharmaceutical Sciences, Nursing, Dental Medicine, and Nutrition Sciences courses being the respondents who most highlighted this fact. Concerning knowledge and ability to identify and, in addition, report potential suspected ADRs, the responding students of Pharmacy, Pharmaceutical Sciences, Nursing and Dentistry also felt most prepared.

On the other hand, a minority (18%) were familiar with pharmacovigilance resources for clinical use and said they had not participated in educational events in which they would increase their knowledge about pharmacovigilance. However, 84% were interested in participating in these kinds of events.

### Teaching-learning perceptions about the disciplinary area of ​​pharmacovigilance

Most students (77.4%) reported that there were contents related to pharmacovigilance in their course, specifically those in the following courses: nutrition sciences, pharmaceutical sciences, nursing, pharmacy, medicine, and dental medicine. However, a minority (24.3%) affirmed that more specific content on this theme had been presented in their courses, as is the case, for example, of *Portal RAM* (Adverse Reaction Notification Portal in Portugal) and the Portuguese Pharmacovigilance System, among others.

### Perceptions about the importance of pharmacovigilance and given global pharmacovigilance training

**Fig. 3:**
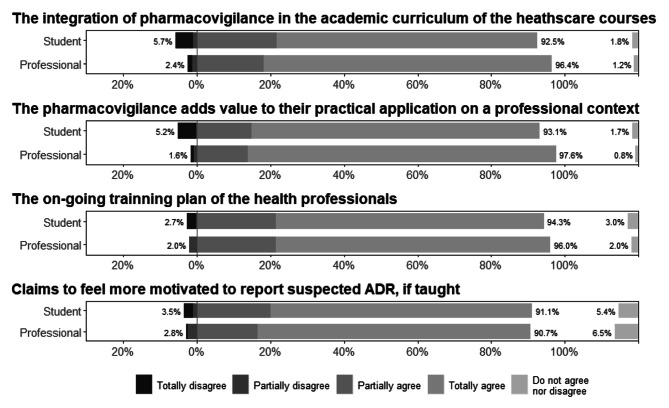
Perceptions about the importance of pharmacovigilance

Using a Likert scale, from *strongly disagree* to *strongly agree*, participants were asked to check the contents presented in Fig. 3; the answer *totally agree* was found to be the most prevalent in both groups of respondents.

Figure 3 shows that 70.7% of the students, as well as 78.1% of the professionals, fully agreed that this subject should be part of their curricular plans, and those who attended courses in Pharmacy, Pharmaceutical Sciences, Nursing, Dentistry, Oral Hygiene, and Medicine had a higher percentage of positive responses.

Most students and professionals, especially those in Pharmacy, Oral Hygiene, and Dental Medicine courses, showed interest in the integration of pharmacovigilance in the academic curriculum of healthcare courses and the ongoing training plans for health professionals. In addition, the majority completely agreed that pharmacovigilance adds value to their practical application in a professional context and claimed to feel more motivated to report a suspected adverse reaction if taught.

More than half of the students (54%) and professionals (66%) reported that they completely agreed with the inclusion of pharmacovigilance in curricular units of courses in health. Regarding the pharmacovigilance approach in continuing education plans for health professionals, agreement was also evident.

### Barriers and facilitators to spontaneous reporting of adverse drug reactions

**Fig. 4:**
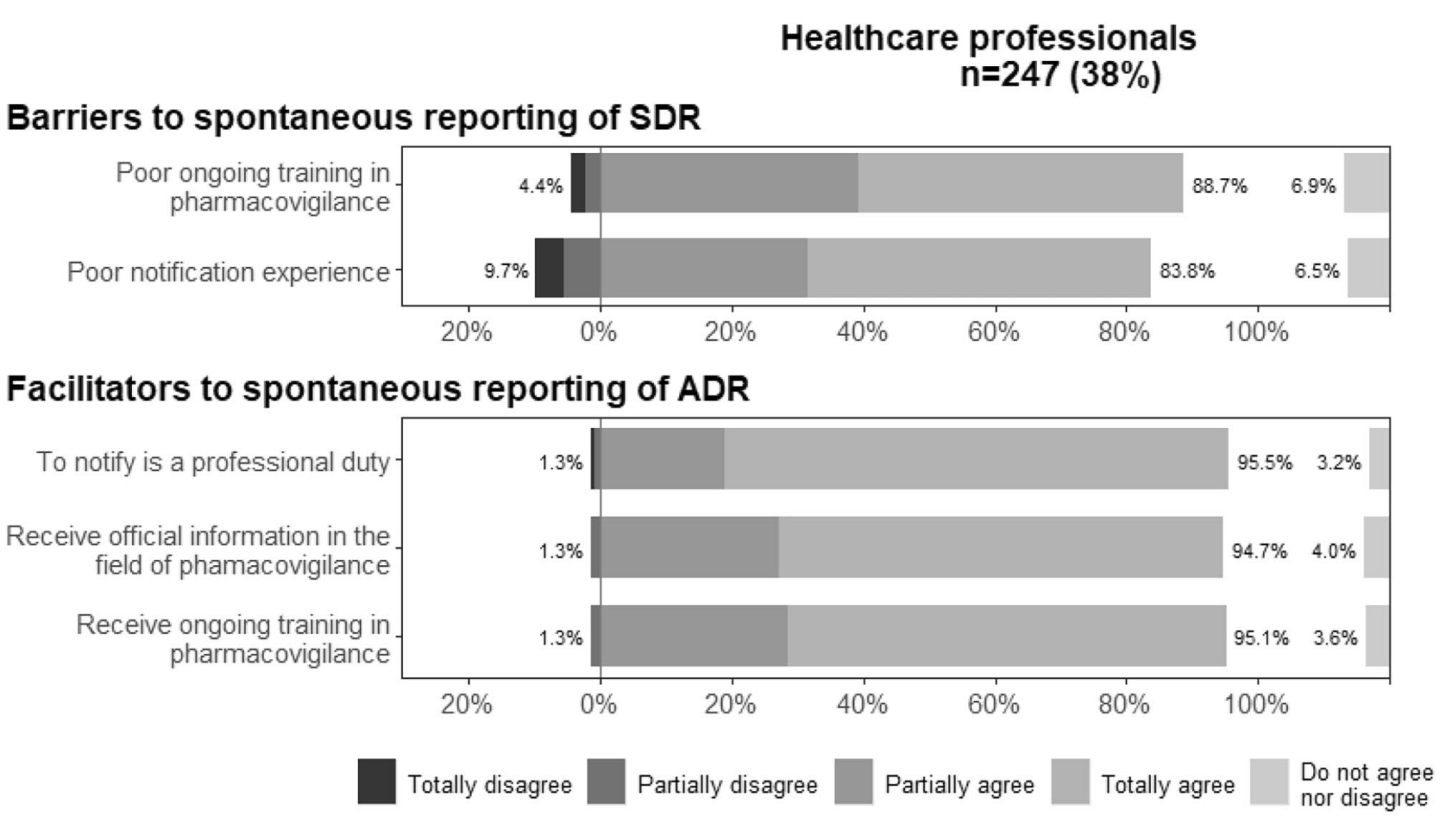
Barriers and facilitators to spontaneous reporting of adverse drug reactions identified by professionals

This section was answered only by health professionals. The main barriers noted by the professionals were poor ongoing training in pharmacovigilance and poor notification experience. The main facilitators noted by the professionals were *“to notify is a professional duty”*, *“receive official information in the field of pharmacovigilance”*, and *“receive ongoing training in this area”*. Another facilitator identified by healthcare professionals at the end of the questionnaire was the simplification of the notification process since they had checked ADR notifications for vaccines against coronavirus disease 2019 (COVID-19) on a site with several network failures in which notifications did not appear, wasting time, so a simplified process would be desirable (Fig. 4).

## Discussion

The low presence of pharmacovigilance in the curricular plan of courses in health may contribute to the apparent lack of knowledge, both of current students and of health professionals, who constitute a preponderant element in this field and, consequently, constitute a relevant cause for underreporting. Thus, the insufficient knowledge demonstrated regarding this theme can be improved through continuous professional development programs, reinforcing theoretical and practical knowledge in the pregraduate curriculum of the different courses in this area [6]. Furthermore, postgraduate training should be further developed through the creation of more programs, be they accredited courses/webinars, workshops [6], or private training, by, for example, INFARMED, I.P. (National Authority for Medicines and Health Products), to promote them [22]. Finally, other indicators of future perspectives were highlighted, namely, the interoperability of systems and the need for articulation among primary, hospital and community pharmacies. In this sense, it is essential to integrate pharmacovigilance and spontaneous reporting of ADRs into each patient’s individual care plan. In this sense, the importance of pharmacovigilance teaching-learning is envisaged, with emphasis on *“learning by doing”*, integrating pharmacovigilance in a real context, from the point of view of researchers, health professionals and citizens in general [23].

The Portuguese population has low health literacy, which constitutes a challenge for reports made by citizens in general [4, 9]. This fact is evidenced by the number of notifications made directly by the population, so it is essential to take measures and implement initiatives to overcome these difficulties and promote the growth of the number of notifications made directly by patients [4, 9]. For example, when citizens have a chronic disease, it becomes even more essential to enable them to report, as they can be active and informed partners in the management of their disease and its therapy [12]. The duty of health professionals to motivate patients to report suspected ADRs they experience directly to the pharmacovigilance system is also relevant [13]. Therefore, a positive attitude on the part of the health professional regarding healthcare providers’ identification and reporting of suspected ADRs is crucial for the pharmacovigilance system to work [14, 15].

Thus, training in pharmacovigilance becomes essential, not only because ADRs are an important health problem and contribute to the increase in the number of hospitalizations but also because of the morbidity and mortality rates, as mentioned above. Therefore, if drug safety is not considered adequate, there can be serious consequences for the patient, but if ADRs are reported, drug safety can be significantly improved [9, 18]. Effectively, since the World Health Organization report of 1972, the discipline of pharmacovigilance has developed as a fundamental part of the clinic characterized above all by its dynamics over time. However, more is needed to integrate the discipline into clinical practice, as well as health policy [3].

In general, both nationally and internationally, the curricula of higher education courses have shown little focus on the topic of pharmacovigilance [24–28], which may eventually justify the low involvement of healthcare professionals in reporting suspected ADRs [29].

According to the literature, at an international level, cross-sectional studies of pharmacovigilance in higher education students in the health area indicate that teaching in this area has been little contemplated and deserved little attention in the curricula of these courses. Thus, it is mentioned that early and frequent exposure to pharmacovigilance concepts may be fundamental, pointing out the fact that the professors who teach a curricular unit in health courses should improve students’ knowledge of pharmacovigilance, both in the syllabus taught in class and during the internship period [26, 30]. It is therefore important and urgent to improve and innovate basic training in terms of pharmacovigilance, particularly for higher education students in health courses [27, 31]. It is expected that teaching, not only with a more practical aspect but also in a previous phase of sedimentation of integrated knowledge, will allow students to understand the learning result in their future activity [32], both in terms of the impact on individual health and collective health, both in terms of generating knowledge and information over time. Thus, students will increase their knowledge and awareness of the area under study [32], but they will also be able to help current health professionals progressively develop pharmacovigilance activities [33]. It is also known that *“learning by doing”*, meaning teaching and raising awareness of this topic using real-life situations, can be more effective than exclusively theoretical teaching, as it enhances the acquisition of skills [27, 33]. Pharmacovigilance interventions that are effective range from short lectures/workshops and more innovative clinical experiences [6, 34] regarding the reporting and assessment of suspected ADRs [32], as well as repeated pharmacovigilance training during internships [35]. Another identified problem is the translation from education on pharmacovigilance to practice. Research has shown that this transition is still not adequate and that reporting practices could be further increased by using user-friendly methods, such as electronic reporting and educational interventions [6, 23].

Reumerman et al. (2021) also presented an initiative that was low cost and saved student time. The project consisted of creating a team made up of medical students to detect, manage and report serious and unknown ADRs in hospitalized patients, which resulted in an increase in the number of suspected ADRs reported in the hospital. This approach was considered viable, as there was additional care for the patients. Students acquired basic pharmacovigilance skills and knowledge and contributed to the reporting of suspected ADRs by health professionals, becoming an asset to all parties involved [36].

The lack of training and the low level of knowledge, skills, and attitudes in this area from several professionals, such as doctors, pharmacists, nurses, and dentists [37–39], were identified, as well as the lack of skills of health professionals in the field of pharmacovigilance [34, 40].

Curriculum plans in place in higher education must provide adequate skills, which ensure correct prescription, dispensing and administration, as well as safe use of medication. Clinical assessment skills should include ADRs as a differential diagnosis, obtaining an accurate therapeutic history, basic individual causality assessment, and informing patients about possible adverse drug effects [41].

Recent studies aimed at assessing the knowledge of health professionals about communications about medication safety, as well as the preferred sources and means of communication, were conducted in some European countries within the scope of the Strengthening Collaboration for Operating Pharmacovigilance in Europe project (SCOPE) Joint Action. In some countries, the results of this project revealed that certain communication tools, more precisely, educational materials, are not as recognized by health professionals and prefer information from health authorities or professional organizations. Other sources, such as websites or newsletters and medical journals, were also considered relevant, and there was an opportunity to explore their use to disseminate information about drug safety [42].

It is known that health professionals still have limited knowledge of pharmacovigilance and how to report suspected ADRs [16, 30, 43], with only a few educational interventions having lasting effects on this knowledge [31]. However, a study implemented at the Pharmacovigilance Unit of the North showed that the educational interventions carried out made it possible to increase the number of notifications by health professionals, namely, doctors and pharmacists [9]. Future professionals should therefore acquire an adequate set of skills in the field of pharmacovigilance to prescribe, distribute and monitor the use of drugs in a rational, safe, and effective way [31, 44]. In addition, together with the fact that “the unexpected is where discovery begins”, the importance of preparing these professionals for the identification of unexpected risk factors in the detection of ADRs and pharmacological therapy is mentioned [45]. Although pharmacovigilance is increasingly based on observational studies, a crucial source of evidence for unexpected drug effects is reporting cases of daily clinical experience [46].

In this context, the teaching of pharmacovigilance to future health professionals is strongly recommended [26, 47, 48]. According to May et al. (2014), within the scope of pharmacovigilance, there are two educational dimensions of the health professional that must be articulated to lead to the notification of suspected ADRs: basic knowledge, which every professional must possess, and advanced knowledge aimed at those who are professionally dedicated to pharmacovigilance, whether they belong to regulatory authorities, regional pharmacovigilance units or the pharmaceutical industry [49]. Therefore, for health professionals to become active vigilantes, when drug ADRs are suspected, knowledge and understanding of the benefits of this practice are essential [50]. A study coordinated by Adenuga et al. (2020) also alludes to the need for continuous professional development in pharmacovigilance, which should be strengthened in all hospitals. The meetings of the Pharmacy and Therapeutics Committee should be educational and motivational to lead to an increase in notifications of any type of ADR by all health professionals [51].

Thus, curricula for health courses should prioritize training on pharmacovigilance [3, 26], as education, training and access to reliable information are possible approaches to increasing awareness and interest in the safety of medication on the part of health professionals [7, 51]. It will be easier to promote, among health professionals, a greater sensitivity to pharmacovigilance when a culture of multidisciplinary thinking is employed [3, 52]. Notably, underreporting, which persists, may be related to a lack of knowledge about how and what to report [6, 53]. Therefore, by reinforcing theoretical and practical knowledge [30] in the curriculum of health courses and through continuing professional development programs, these knowledge gaps can be filled [4, 47].

Our study had limitations identified in the indirect analysis related to the obtained sample, selection bias, the syllabi of the curricular unit forms summarized, and fulfilment of the programs described. In the direct analysis, potential limitations were selection bias, information bias, social desirability bias and the representativeness of the sample. Regarding the collection of data on the syllabus by the curricular unit forms of the preselected courses, it should be noted that this was only carried out in public higher education institutions due to the open access to form data, which are available on the websites of the respective educational institutions. In case of public unavailability of the files, the curricular unit forms were requested by direct contact, more precisely, via email to the course directors.

## Conclusions

Since few institutions are teaching programmatic content regarding pharmacovigilance in the different healthcare courses and given the questionnaire results, it seems there is a need for a wider reflection regarding further training and constant update of practicing professionals as well as in the diverse health institutions. The results highlight the need for greater reflection on the ongoing training of professionals in service, as well as the commitment to the creation of curricular units that integrate pharmacovigilance in health courses.

The present study is pioneering, aiming at the identification, review, description, and curricular characterization of several courses in the health area in Portugal regarding the contents of pharmacovigilance, with a view to a better knowledge of undergraduate training.

This innovative research project in Portugal included a pharmacovigilance curricular review in Portugal and a review of the knowledge of students and health professionals.

The insufficient training and knowledge, underreporting, and the low presence of pharmacovigilance contents in the curricular plans of courses in health can and should be improved through the reinforcement of pregraduate training, postgraduate training and continuous formation, i.e., workshops.

The results highlight the importance of teaching and learning pharmacovigilance, emphasizing learning by doing, real context integration and a community-oriented approach.

Since few institutions teach syllabi related to pharmacovigilance in different health courses, educational measures should be taken both for higher education students and health professionals. It is advisable to create a pharmacovigilance curricular unit that is able to adjust to the basic training of each type of professional to prepare each student for their daily practice.

## Data Availability

The database and analyses for the present study are available from the corresponding author upon reasonable request.
